# Non-biopsy Strategy for the Diagnosis of Celiac Disease in Adults: A Narrative Review

**DOI:** 10.5152/tjg.2024.24092

**Published:** 2024-08-01

**Authors:** Herbert Wieser, Carlo Soldaini, Carolina Ciacci

**Affiliations:** 1 Hamburg School of Food Science, Institute of Food Chemistry, University of Hamburg, Hamburg, Germany; 2 Department of Medicine, Gastrointestinal Unit, Surgery and Dentistry “Scuola Medica Salernitana”, University of Salerno, Salerno, Italy

**Keywords:** Celiac disease, diagnosis, non-biopsy strategy, anti-transglutaminase IgA

## Abstract

Celiac disease (CeD) diagnosis is a complicated process, requiring a multi-step procedure and a high level of clinical knowledge. Some scientific societies, mainly from Europe and North America, have proposed appropriate guidelines for the diagnosis and management of CeD. Since duodenal biopsy is particularly challenging for children, guidelines of the European Society for Pediatric Gastroenterology, Hepatology, and Nutrition, presented in 2012 and updated in 2020, have made it possible to avoid the biopsy in symptomatic pediatric patients with high levels of IgA anti-transglutaminase. Several parallel, similar studies in adults support the non-biopsy strategy. However, several pros and cons exist in applying such a strategy. The present narrative review reports the current evidence and the implication of omitting biopsy in the diagnosis of CeD in adults.

**Main Points**

- There is growing evidence in scientific literature supporting a non-biopsy approach for the diagnosis of Celiac disease in adults.
- The implications, whether positive or negative, of adopting such a strategy are being debated in the scientific community.
- This narrative review presents an overview of the current literature regarding the serologic diagnosis of celiac disease in adults, without histological examination of the duodenum.

## Introduction

Celiac disease (CeD) is an immune-mediated illness of the upper small intestine precipitated by gluten consumption of wheat, rye, and barley products, in genetically predisposed individuals. The general aspects of CeD have recently been reviewed by Lebwohl and colleagues.^1^ Summarily, the estimated global prevalence of diagnosed CeD is 1% of the overall population.

However, CeD is one of the most underdiagnosed disorders worldwide: when extrapolating current population data, there are around 22 million women and 16 million men with undetected and thus untreated CeD.^2^ Particularly, asymptomatic CeD and CeD with solely extra-intestinal manifestations are at high risk of remaining undetected. CeD is genetically linked with the human leukocyte antigen (HLA) class II alleles HLA-DQ2 and -DQ8 at the major histocompatibility complex. Clinically, CeD can cause gastrointestinal symptoms such as abdominal pain, vomiting, and chronic diarrhea as well as extra-gastrointestinal symptoms that include nutritional deficits, bone diseases, reproductive problems, and neurological and psychiatric disorders. Pathologically, CeD can lead to an inflammatory deterioration of the small intestinal mucosa (“flattened mucosa”) along with specific antibodies directed against gluten proteins (antigens) and tissue transglutaminase (autoantigen). Lifelong strict observance of a gluten-free diet (GFD) is currently the sole viable therapy for CeD.

The diagnosis of CeD is a complicated process that calls for several steps and advanced clinical expertise. Appropriate criteria for the diagnosis and treatment of CeD.^3^ have been proposed by a number of scientific associations, primarily from North America and Europe. To summarize, the diagnostic protocol should contain the following steps: (1) Clinical history and symptomatology; (2) serology; (3) small intestinal histology; (4) response to the GFD; and, eventually, (5) genetic status. Sadly, however, distinct issues confront every stage of the diagnostic protocol. For example, serological testing has been so far restricted by different sensitivity, specificity, and overall performance of assays that are applied in the diagnosis and are targeted on endomysium (EMA), tissue transglutaminase (TGA), and deamidated gliadin peptides antibodies (DGPA).^4^ Furthermore, up to 10% false-negative diagnoses of CeD are caused by seronegative CeD.^5^ Additionally, as most assays target antibodies of the IgA class, they do not help evaluate individuals who have an IgA deficiency (about 7% of patients with CeD).

The invasiveness of the procedure, the variation in pathologists’ findings, the low reproducibility and agreement in the grading of villous atrophy, and finally yet importantly, the excessive costs are the main drawbacks of histological examination of the duodenum.^6^ Since duodenal biopsy is particularly challenging for children, guidelines of the European Society for Pediatric Gastroenterology, Hepatology, and Nutrition (ESPGHAN), presented in 2012 and up-dated in 2020, have made it possible to bypass the duodenal biopsies in pediatric symptomatic patients with high levels of IgA TGAs (greater than 10 times the upper limit of normal (ULN)), positivity to IgA EMA, and presence of HLA-DQ2/8 genes (“triple criteria”).^7,8^ The “no-biopsy approach” regulation is still controversially discussed and not yet implemented in most parts of the world. The advantages and disadvantages of a biopsy-avoiding diagnostic method and the contrast between pediatric and adult regulations have been discussed by Raiteri et al^3^ and Reilly et al.^9^

It is still debated whether CeD in adults can be identified solely through serological testing. Therefore, current international guidelines still recommend a duodenal biopsy to confirm adult CeD diagnosis.^10-12^ A recent systematic review and meta-analysis, including 18 international studies and more than 12 000 adult participants, substantiated that selected adult patients with IgA TGA ≥10xULN and a moderate to high pre-test probability of CeD can be diagnosed without undergoing invasive endoscopy and duodenal biopsy.^13^ The goal of this narrative review was to present an overview of the current literature regarding the serologic diagnosis of CeD in adults, without histological examination of the duodenum.

## Materials and Methods

A thorough literature search was separately conducted electronically in the PubMed database by the 3 authors (C.S., H.W., C.C.) for articles published in English between 2013 and 2023, using “c(o)eliac disease” as a main search term, in variable conjunction with “diagnosis,” “biopsy,” “adults,” and “anti-tissue-transglutaminase.” Supplementary resources were obtained from personal CeD archives and by cross-referencing the identified articles.

### Initial Investigations on Serological Diagnosis of Celiac Disease in Adults

#### Anti-Gliadin Antibodies and Anti-Endomysial Antibodies:

First CeD-specific serological tests, presented in the 1970s and 1980s, used anti-gliadin antibodies (AGA) as a target reactant, but their sensitivities and specificities were fairly poor. These problems were solved by the introduction of EMA tests based on the findings by Chorzelski et al.^14^ The diagnostic value of IgA AGA and IgA EMA assays in adults with histologically confirmed CeD was first reported in 1996.^15^ Serum samples collected from 144 adult Swedish patients who had duodenal biopsy and did not have contemporaneous dermatitis herpetiformis or an IgA deficit were tested for both IgA EMA and IgA AGA. Nineteen patients (13%) had biopsy-verified CeD. The sensitivity and specificity of IgA EMA were 74% and 100%, respectively. The diagnostic accuracy was 97%, and the positive and negative predictive values (PPV and NPV) were 100% and 96%. In contrast, IgA AGA had PPV and NPV of 28% and 96%, respectively, with a diagnostic accuracy of 71%. The authors then suggested that in symptomatic individuals who have IgA EMA, a small intestinal biopsy is not required to diagnose CeD. However, due to a NPV of 96%, some symptomatic adults lacking EMA would not be correctly diagnosed without a duodenal biopsy.

### Anti-transglutaminase Antibodies

Dieterich et al, in 1997, identified tissue transglutaminase (also known as transglutaminase 2) to be the autoantigen of CeD, which allowed the development of ELISA-based TGA tests, a milestone in the history of serological testing.^16^ In subsequent studies, the association of EMA and TGA titers with duodenal histology was investigated. One hundred eighty-one Italian patients (50 adults) referred to the clinic for the suspect of CeD underwent EMA and TGA testing and duodenal biopsy.^17^ The overall accuracies of TGA and EMA testing were 92.8% and 93.4%, respectively. The NPVs of TGA and EMA were 97.2% and 87.2%, respectively. The EMA and TGA had mean positive likelihood ratios of 7.48 and 3.89, respectively. In patient populations with an estimated prevalence of more than 75% for CeD, such as those exhibiting characteristic CeD symptoms, serial testing would yield a post-test probability greater than 99%. The findings revealed that repeated testing using TGA and EMA may, in certain circumstances, eliminate the need for biopsies to confirm the diagnosis of CeD.

To determine the TGA level giving a PPV for CeD of 100%, 146 adult patients with TGA levels >10 U/mL were included in a retrospective study from the UK.^18^ Of these individuals, 139 had CeD and the Marsh classification of the small bowel biopsies was between 2 and 3C. Seven patients had normal biopsies and TGA results between 10 and 30 U/mL. Each patient with TGA levels >30 U/mL, i.e., 10 × ULN, had characteristic small intestine mucosal lesions. The authors proposed changing the diagnostic criteria to exclude the requirement for small intestinal biopsies in patients with such high TGA levels.

Donaldson and coworkers determined whether high serologic IgA TGA is exclusively associated with CeD.^19^ Out of the 1882 US children and adults who had their IgA TGA tested, 208 had an IgA TGA ≥100 U/mL. Among them, 76 patients, consisting of 28 children and 48 adults, also underwent duodenal biopsies. 73 (96%) of those 76 patients had a biopsy that revealed villous atrophy (Marsh 3). The Marsh histology of the other 3 patients was moderate. In conclusion, in both adults and children, the setting of Marsh 3 duodenal histology is almost exclusively associated with IgA TGA ≥100 units.

To evaluate TGA’s predictive value for villous atrophy in Spanish subjects, a prospective analysis was conducted on 324 CeD patients (97 children and 227 adults).^20^ Upper gastrointestinal endoscopy and IgA TGA assay were conducted at the time of diagnosis. Compared to children, adults had significantly less severe histopathology (26% vs. 63%, *P* < .0001) and lower TGA titers. In every group, there was a significant correlation (*P* < .0001) between the levels of TGA and Marsh type. Notably, TGA was the only independent predictor of Marsh 3 lesions; age and clinical presentation type were not. The receiver operating characteristic curve’s maximum area under was obtained with a cutoff value of 30 U/mL TGA. Without biopsy, up to 95% of children and 53% of adults would receive the right diagnosis, according to this cutoff point’s predictive value. Consequently, highly elevated TGA titers may be adequate for diagnosing CeD in children. On the other hand, the findings showed that duodenal biopsy cannot be avoided in adults.

To evaluate the efficacy of multiple serological tests, separately and combined, 679 adult Argentinians with high (161) or low (518) risk of CeD, underwent duodenal biopsies in addition to serological testing.^21^ Blood samples were analyzed using 6 enzyme-linked immunosorbent assays (ELISAs) that detected TGA or DGPA. Celiac disease prevalence was 3.3% in the low-risk subset and 39.1% in the high-risk population. Using assay combinations, e.g., the new DGP/tTG screen assay, in 92% of cases, in both groups, a diagnosis of CeD could be made or ruled out without a biopsy.

In order to establish a TGA cutoff value with a high positive likelihood ratio that indicates duodenal atrophy, a sample of 945 adult Italian patients with suspected CeD was retrospectively studied.^22^ The Marsh classification was used to classify the duodenal histology. The cutoff thresholds of TGA as a predictor of villous atrophy were determined using sensitivity, specificity, and positive likelihood ratio analysis. The findings revealed 100% specificity and ∞ positive likelihood ratio for duodenal atrophy when adopting a cutoff value of TGA 5 times higher than the ULN. In one-third of the cases, a biopsy might be avoided with this limit.

A study that retrospectively analyzed results from 2477 US patients who had serology tests for CeD, was conducted to determine the amount of times the adult patients with positive serological results were referred for small-bowel biopsies.^23^ Biopsy samples were analyzed by pathologists according to the Marsh classification. A duodenal biopsy was performed on 238 patients (39%) out of the 610 patients (25%) who had abnormal results from serology tests. 50 (21%) of the participants reported biopsy results that were in line with CeD (Marsh 3). With a 2% false-positive rate, patients with CeD were identified by tiers of IgA TGA more than 118 U/mL. For CeD, titers ranging from 21 to 118 U/mL provided an 83% PPV when combined with an EMA dilution titer of 1 : 160 or higher. Immunoglobulin A TGA levels less than 20 U/mL, combined with an EMA dilution titer less than 1 : 10, resulted in an NPV of 92% for CeD. In conclusion, in the absence of a duodenal biopsy, adult symptomatic patients with CeD can be identified by serum levels of IgA TGA higher than 118 U/mL or 21 to 118 U/mL in conjunction with an EMA dilution titer of 1:160 or above.

To determine whether intestinal biopsy could be replaced by serological testing in both children and adults, Bürgin–Wolff and colleagues investigated serum samples from 149 pediatric and adult patients with CeD and 119 controls, all of them having duodenal histology.^24^ Every serum sample underwent analysis for AGA, DGPA, TGA, and EMA. A combination of all 4 tests yielded PPV and NPV of 99% and 100%, respectively, and a likelihood ratio positive of 86 with a likelihood ratio negative of 0.00. Even after excluding the EMA values, the resulting PPV and NPV were 99% and 98%, while the likelihood ratio was positive at 87 and negative at 0.01. Therefore, in a large percentage of patients (78%), a combination of 3 or 4 tests allowed for the diagnosis or exclusion of CeD without the need for an intestinal biopsy. Exclusively those patients with discordant antibody results (22%) were found to require a biopsy.

In summary, since the 1980s, serological testing of EMA, TGA, and DGPA levels, alone or in combination, became a standard method as part of the diagnostic procedure for both children and adults with suspected CeD. A number of investigations could demonstrate a close relationship between the level of serum TGA and duodenal damage (Marsh classification). As a result, ESPGHAN presented guidelines that allow diagnosis of CeD in children without duodenal biopsy when the triple criteria are fulfilled. In contrast, although most studies cited here (7 out of 8) have demonstrated that adults can also avoid a duodenal biopsy when specific serological preconditions exist, this approach has not been officially recommended. Therefore, adequate investigations on adults, regarding the diagnostic accuracy according to ESPGHAN guidelines for children, were continued as shown in the following section.

### Diagnostic Accuracy According to European Society for Pediatric Gastroenterology, Hepatology, and Nutrition Guidelines

To investigate the applicability of the ESPGHAN criteria, 234 Italian adults, with elevated TGA levels, EMA positive and genetically predisposed, had upper endoscopic examination with duodenal biopsies.^25^ Receiver operating characteristic (ROC) curves were used to determine optimal TGA cutoff levels. Mean TGA levels were 71.1 U/mL, and mean normalized levels were 14.8 × ULN. Partial and total villous atrophy was present, respectively, in 36% and 55% of patients, TGA levels significantly correlated with histology (*P* < .001). Applying the ESPGHAN criterion (≥10 × ULN) resulted in 97.7% PPV. Receiver operating characteristic curve analysis revealed an ideal cutoff of ≥16 × ULN with a PPV of 98.9%.

A retrospective analysis of 270 adult CeD patients from the UK, examined by serum TGA levels and small intestinal biopsies, revealed that a significant percentage of adults can be accurately diagnosed with CeD just by serology, utilizing IgA TGA.^26^ When the antibody cutoff exceeded the value of 45 U/mL (>8 × ULN + 2 SD), the PPV for the diagnosis of CeD was 100%; 40% of cases were above this cutoff. These results showed further proof that a biopsy is not always necessary for the diagnosis of CeD in adults.

In an Indian study, patients with serum TGA levels >15 U/mL and who underwent biopsy were selected for further evaluation.^27^ Of the 731 patients taken into consideration, 261 had TGA levels less than 100 U/mL and 470 had levels greater than 100 U/mL. Compared to the lower historical grade Marsh 1, which had a mean TGA level of 109 U/mL (>7 times the cutoff value), Marsh 3 had a mean TGA level of 187 U/mL (>12 times the normal cutoff value). With a TGA threshold of 70 U/mL, sensitivity was 83.9%, specificity was 56.1%, and the overall precision was 77.7%. The authors came to the conclusion that, in symptomatic individuals with elevated TGA levels (>70 U/mL), a biopsy is not necessarily required to identify CeD.

To assess the accuracy of serology-based criteria according to ESPGHAN guidelines for children, 274 adult patients with biopsy-proven CeD were serologically evaluated in 3 clinical centers in Finland.^28^ All in all, 90 subjects fulfilled the triple criteria (TGA >10 × ULN, positive EMA, and appropriate genetics) giving the criteria a PPV of 100%. In triple-positive participants, no histological abnormalities other than CeD were seen in their biopsies. Altogether, of the 274 recently diagnosed patients, one-third might have avoided biopsies. In conclusion, adults who meet the triple criteria for CeD could be safely and accurately diagnosed with CeD.

A Turkish study also evaluated adult patients according to the ESPGHAN criteria.^29^ A cohort of 39 subjects with symptoms of CeD underwent serological tests, standard duodenal biopsy, and HLA typing. The results revealed that 21 subjects had biopsy-confirmed CeD. By ROC curve analysis, the best diagnostic specificity result of IgA TGA was detected with a threshold value >32.8 U/mL. Accordingly, 15 out of 21 adult CeD patients (71%) could be diagnosed without biopsy, when clinical symptoms, serological tests, and HLA typing were in accordance with CeD criteria.

Studies of 134 adult Saudi patients with duodenal histology ranging from Marsh 0-3c showed that the IgA TGA serology group with ≥20 U/mL constituted the major part of the patients, that was, 121 (90.3%) vs. 13 (9.7%) with <20 IU/mL.^30^ The authors concluded that in patients with suggestive symptoms, positive TGA tests (with higher titer than 5-10 times the ULN) could be applied to diagnose CeD without the need for a biopsy. The authors suggested that patients who do not exhibit clinical improvements on a GFD nevertheless require biopsies.

A 7-year retrospective review by Johnston and coworkers selected 433 patients with positive TGA-IgA.^31^ Overall, 98 individuals (23%) met the high titer requirements for a non-biopsy protocol, which would have lessened the endoscopic workload on the service. A 95% vs. 75% histological confirmation of CeD was obtained with a high TGA-IgA titer compared to a low titer (*P* < .01). The addition of EMA analysis had almost no impact on these predictive rates.

A retrospective study from New Zealand evaluated 144 TGA-positive adult patients, among them 86 patients (60%) had CeD (Marsh 1-3).^32^ While the ROC curve was used to evaluate sensitivity and specificity, linear models were used to establish the link between titers and disease. For CeD in the dataset, an IgA TGA titer cutoff value of 150 U/mL had 100% specificity and 70% sensitivity for this patient population. Regardless of age, gender, or ethnicity, the authors concluded that an IgA TGA titer ≥10 × ULN is a very reliable predictor of CeD.

To test the performance of 4 commercial TGA assays, serum samples of 836 Finnish adults with family risk or either clinical suspicion of CeD were analyzed in a multicenter retrospective investigation.^33^ A total of 137 patients with clinical suspicion and 85 patients with family risk had biopsy-confirmed CeD. PPV for 10 × ULN was 100% in each TGA test. Using the assays’ own cutoff value (1 × ULN), the PPV ranged from 84% to 100%. Thus, serology-based diagnosis of CeD was accurate in adults using different commercial kits and pre-test probabilities applying 10 × ULN. The outcomes additionally suggested that a lower ULN threshold could be appropriate for the biopsy-omitting strategy.

In order to determine the diagnostic yield and the predictive capacity of a 10-time rise in serum IgA TGA levels for detecting intestinal damage in adult patients from different international centers, the following 3 cohorts were studied.^34^ Seven hundred forty CeD patients were evaluated in a specialized CeD clinic for cohort 1; in cohort 2, 532 patients with low suspicion for CeD were referred to undergo an upper gastrointestinal endoscopy; and 145 patients with elevated TGA titers from various international sites for Cohort 3. Using the Marsh 3 histology as a reference, the efficacy of an IgA TGA titer of ≥10 × ULN for the diagnosis of CeD was determined. For IgA TGA levels of ≥10 × ULN, the sensitivity, specificity, PPV, and NPV in cohort 1 were 54.0%, 90.0%, 98.7%, and 12.5%, respectively; in cohort 2, the corresponding values were 50.0%, 100.0%, 100.0%, and 98.3%, and in cohort 3, the corresponding values were 30.0%, 83.0%, 95.2%, and 9.5%. These results showed that IgA TGA titers of ≥10 × ULN have a strong predictive value at identifying adults with intestinal changes diagnostic of CeD.

The results of endoscopic biopsies and serological tests of 269 Turkish adults were retrospectively evaluated by Baykan et al.^35^ Patients with villous atrophy had considerably higher TGA values (*P* < .001), and there was a positive correlation between TGA values and mucosal injury (*P* < .01). When the threshold value of TGA was 100 U/mL (>10 × ULN), the sensitivity was 71.6%, specificity was 100%, and the PPV was 100%. The sensitivity was 100% and the specificity was 99.5% when the cutoff value was set at 29.42 U/mL. According to the ESPGHAN guidelines, this study supports the use of a biopsy-free approach in adults.

The study by Hoyle and coworkers intended to ascertain if the recommended TGA-IgA ≥10 × ULN is a safe diagnostic tool for UK adult and adolescent patients and whether a biopsy would prevent the identification of any significant co-patology.^36^ Altogether, 1037 out of 1429 patients with positive serology proceeded to biopsy, of which 796 out of 1037 patients (76.8%) were diagnosed as CeD. A total of 320 out of 322 patients (99.4%) with IgA TGA ≥10 × ULN were diagnosed as CeD giving the cutoff a PPV of 99.4%. During endoscopy, no substantial co-pathology was discovered in these individuals. These findings demonstrated that it is safe to diagnose CeD without a biopsy when employing an IgA TGA threshold of ≥10 × ULN, and that no significant pathology would be overlooked.

In an international study with contributions from different centers, 436 adult subjects with data on serum IgA TGA and duodenal histology were evaluated.^37^ Of the participants, 363 (83%) had positive IgA TGA and 73 (17%) had negative IgA TGA. Following local evaluation, 341 of the 363 individuals who tested positive for seropositivity had positive histology (true positives), and 22 had negative histology (false positives). Of the 73 individuals with seronegativity, 7 had positive histology (false negatives) and 66 had negative histology (true negatives) after local review. The results showed a 93.9% PPV, 90.4% NPV, 98.0% sensitivity, and 75.0% specificity. After 29 discordant cases underwent central reconsideration of duodenal histology, the findings showed a PPV of 95.9%, NPV of 90.4%, sensitivity of 98.0%, and specificity of 81.5%.

When the serological criterion was set at greater multiples of the ULN (*P* < ·0001), the PPV rose, regardless of whether the local or central definition of duodenal histology was used. The authors came to the conclusion that, in individuals with a strong suspicion of having CeD and a high serum IgA TGA, biopsy can be safely avoided in the diagnosis of CeD.

A study from Ireland aimed to determine the relation between TGA ≥ 10 × ULN and histological findings.^38^ In a retrospective analysis, 164 adult patients were evaluated who had raised TGA titers and/or histological changes of biopsies classified according to Marsh. Of these 164 patients, 68 (41%) had TGA titers ≥10 × ULN. Among these, 67 subjects had Marsh 3 and 1 subject had Marsh 2. Altogether, a 98.5% PPV of determining CeD (i.e., Marsh 3) in those with TGA ≥ 10 × ULN was found, which may make the biopsy unnecessary for diagnosis.

In summary, every study included here demonstrated that in the case of individuals with a strong clinical suspicion of CeD and a high serum IgA TGA, a duodenal biopsy could be safely avoided in the diagnostic process; although only local validation of test-specific thresholds will guarantee that this method has a major impact. Including EMA testing in the no-biopsy approach may hinder its implementation (costly, labor-intensive, and high inter-server variability) without having a clear added value.^13^ Patients on a GFD and patients with IgA deficiency should be excluded from the no-biopsy approach. Furthermore, a biopsy is still needed in patients who do not show clinical improvement on a GFD.

Implementing the no-biopsy approach in adults could have benefits not only by avoidance of an invasive, uncomfortable procedure, but also in clinical costs and environmental conditions. According to a study by Shiha and coworkers, approximately 3000 endoscopies for suspected CeD could be avoided each year in the UK.^39^ The National Health Service of the UK may save almost £2.5 million in direct and indirect expenditures annually and lower the endoscopic carbon footprint by 87 tons of CO_2_ by implementing the no-biopsy approach for the diagnosis of CeD in adults.

### Correlation of Serology and Histology

In the era pre-serum TGA discovery, the histological evaluation of the small intestinal mucosa was regarded as the gold standard for CeD diagnosis. The microscopic evaluation of biopsies should consider the following targets: luminal surface, enterocyte thickness, ratio of villous height to crypt depth, and number of intraepithelial lymphocytes. The so-called Marsh–Oberhuber classification (Marsh types 0-4) is mostly used by pathologists to evaluate the mucosal lesions.^40^ Marsh types 2-4 are strong indicators of CeD. Since several years, attempts have been made to correlate the level of serum TGA with the degree of duodenal damage (Marsh types) in adult CeD.

At a single UK center, the association between TGA levels and Marsh 3 histology in the seropositive adult and pediatric population was examined using retrospective laboratory data.^41^ Among 202 seropositive patients with corresponding biopsies, it was possible to define a TGA cutoff with 100% specificity for Marsh 3 histology, at just over 10 × ULN for the method. These results supported the view that high-titer TGA levels had strong predictive value for villous atrophy in adults and children but suggested that decision cutoff values to guide biopsy requirements will require local validation.

In total, 671 adult Italian CeD patients underwent endoscopy with biopsy to perform duodenal histology and blood collection to measure TGA levels.^42^ The degree of duodenal lesions (Marsh types) was compared to the TGA levels/cutoff ratio. The findings showed that the TGA levels/cutoff ratio in type 3c patients was significantly greater than that in type 3b (*P* < .001), 3a (*P* < .001), 2 (*P* < .05), and 0 (*P* < .001) patients. In almost 75% of seropositive persons waiting for a CeD examination, the threshold value >3.6 (sensitivity = 76.8%, PPV = 97.2%) produced the greatest diagnostic performance, avoiding endoscopy with biopsy. The TGA levels/cutoff ratio was acknowledged as an accurate criterion (*P* < .0001).

A descriptive study was conducted on 299 Iranian volunteers to determine the precise level TGA level that can be used to predict CeD in adults with no need for biopsies, as well as to evaluate the link between TGA titer and pathological results.^43^ Analysis of ROC curve was used to find a cutoff point of TGA level for mucosal atrophy. Mean TGA titers were significantly higher in patients graded as Marsh 3 (*P* = .023). Receiver operating characteristic curve analysis showed 89.1% sensitivity for cutoff point ≥76.5 U/mL of TGA. For Marsh ≥2, specificity was 28% and PPV was 91%. In conclusion, there was a linear correlation between increasing TGA level and Marsh type 1 to 3. The specificity of TGA titers more than 200 U/mL was 100% for Marsh >2.

A retrospective study from Saudi Arabia determined the IgA TGA level of 134 adolescents and adults, whose histopathologic investigation of duodenal biopsies revealed Marsh types ranging between 0 (16 cases), 1 (8), 2 (4), 3a (32), 3b (64), and 3c (10).^30^ Among the cases,13.2% had a negative intestinal biopsy and IgA TGA levels ≥20U/mL. A Spearman correlation analysis was carried out to evaluate the connection between Marsh classification and IgA TGA titers. A statistically significant (*P* = .001) moderately positive association (*r*_s_ = 0.4) emerged.

The results of serological tests and endoscopic biopsy of 269 Turkish adults were retrospectively evaluated to estimate the relation between serum IgA TGA levels and Marsh types.^35^ There were no significant differences in TGA titers when compared to mucosal injury, both in a situation of normal mucosa (Marsh 0) and elevated lymphocyte or crypt hyperplasia (Marsh 1-2). When compared to the other groups, the TGA values were considerably greater in the presence of partial or entire mucosal atrophy (Marsh 3a-3c). There were no apparent significant variations in TGA levels across patients with Marsh 3a-3c. Mucosal damage and TGA levels were shown to be positively connected in the Spearman analysis (*r* = 0.60, *P* < .01).

As a result of the most recent studies, histology remains a gold standard in the case of low-titer TGA or discordance with EMA. However, most authors point out that several factors such as the lack of adherence of endoscopists in sampling bulb and second duodenum mucosa,^44,45^ lack of useful information for the pathologists, and incorrect handling/orientation of samples may hamper its utility.^46^

In summary, although large-scale international studies on the correlations between the degree of duodenal damage and levels of serum TGA are needed, errors in sampling, reading and interpreting histology in CeD are a known disadvantage of using histology as a mandatory tool for CeD diagnosis.

### Pros and Contras of the Non-Biopsy Approach

**Figure 1.**
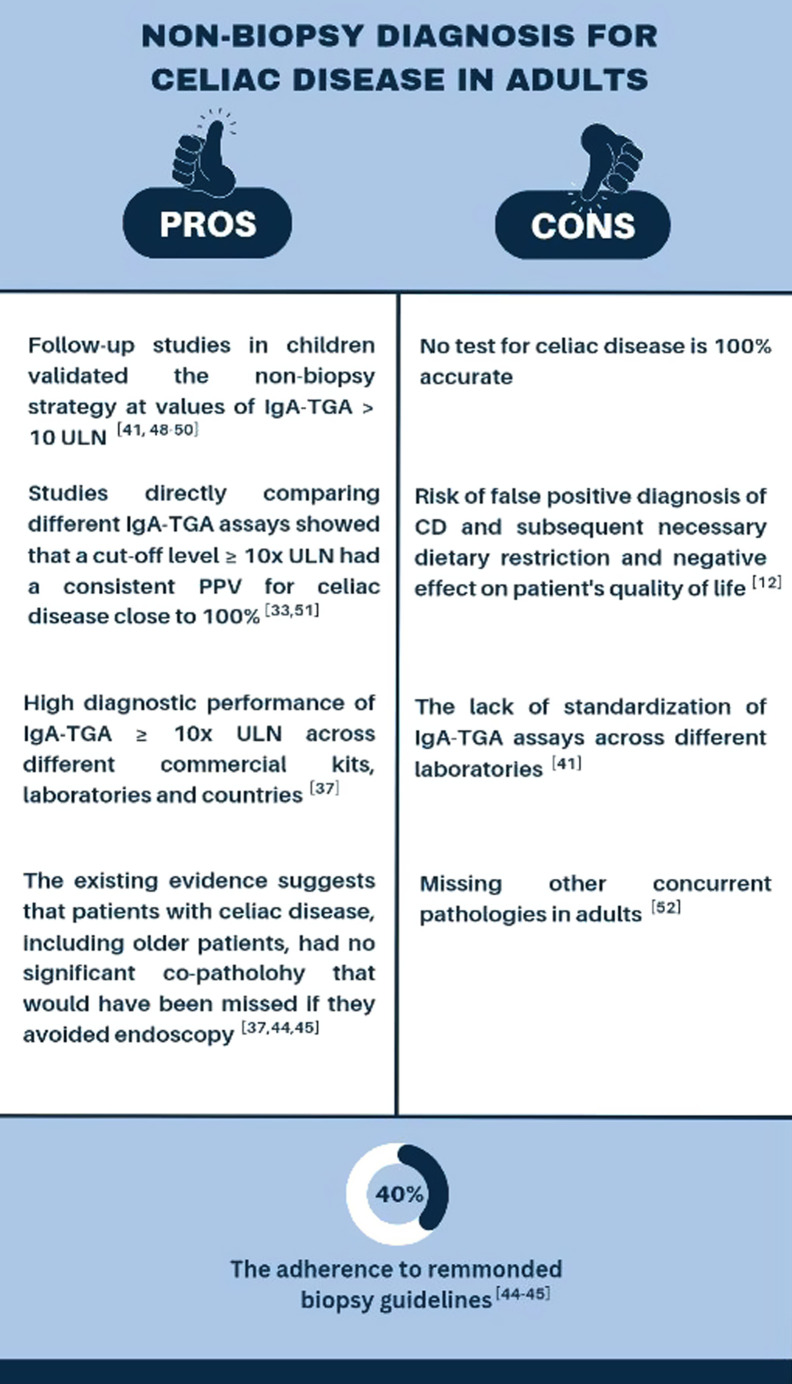
Summary of pros and cons of a non-biopsy strategy for the diagnosis of celiac disease in adults. The adherence to guidelines for correct sampling of duodenal biopsies is low.

Growing evidence supports non-biopsy approaches for the diagnosis of CeD in adults, particularly in scenarios of high titer TGA, and the most recent literature allows the evaluation of pros and cons of avoiding biopsies^47^ (Figure 1).

Among the pros are the follow-up studies in children that have validated the non-biopsy strategy for values of TGA ≥10 × ULN,^41,48-50^ and the studies comparing different IgA-TGA assays consistently showing a near 100% PPV for CeD, using a cutoff level of ≥10 × ULN.^33,51^ This uniformity across various commercial kits, laboratories, and countries enhances diagnostic accuracy and reliability.^37^ The risk of missing other diseases in older patients is relatively low, as demonstrated by studies in which older patients with CeD do not typically exhibit significant co-pathologies that would be missed by avoiding endoscopy.^37,44,45^ These findings support the non-biopsy approach even in older populations, reducing the need for invasive procedures, especially when considering that adherence to recommended biopsy guidelines remains suboptimal, with only 40% of cases following the prescribed protocol.^44,45^

Despite all the above advantages, the non-biopsy strategy is limited to cases showing high TGA positivity. Avoiding endoscopy and biopsy may overlook concurrent pathologies^52^ and, while focusing on CeD, other conditions with similar symptoms may remain undetected, potentially delaying appropriate management. Another issue to consider as a con is that until today, the lack of standardization of TGA assays across laboratories poses challenges,^41^ highlighting the need for standardized protocols and validation procedures. Another possible consequence of omitting the biopsy is that the false-positive or potential CeD diagnoses may increase, carrying risks such as unnecessary dietary restrictions and adverse effects on the patient’s well-being.^12^ Last, we cannot predict from the current literature how the no-biopsy approach would be utilized in general practice.^47^

## Conclusion

In conclusion, while the non-biopsy strategy offers several advantages in CeD diagnosis, including efficiency, consistency, and reduced invasiveness, it also presents non-negligible challenges. Further research is needed to assess the outcome of adopting a non-biopsy strategy for adults. At the moment, the evaluation of pros and cons of each single case will optimize diagnostic approaches and improve patients’ outcomes in CeD management.
